# Nosocomial outbreak caused by the SARS-CoV-2 Delta variant in a highly vaccinated population, Israel, July 2021

**DOI:** 10.2807/1560-7917.ES.2021.26.39.2100822

**Published:** 2021-09-30

**Authors:** Pnina Shitrit, Neta S Zuckerman, Orna Mor, Bat-Sheva Gottesman, Michal Chowers

**Affiliations:** 1 Infection Control Unit, Meir Medical Center, Kfar Saba, Israel; 2 Sackler Medical School, Tel-Aviv University, Tel-Aviv Israel; 3 Central Virology Laboratory, Ministry of Health, Chaim Sheba Medical Center, Tel-Hashomer, Israel; 4 Department of Epidemiology and Preventive Medicine, School of Public Health, Sackler Faculty of Medicine, Tel-Aviv University, Israel; 5 Infectious Disease Unit, Meir Medical Center, Kfar Saba, Israel

**Keywords:** COVID-19, Israel, Delta variant, SARS-CoV-2, Nosocomial

## Abstract

A nosocomial outbreak of SARS-CoV-2 Delta variant infected 42 patients, staff and family members; 39 were fully vaccinated. The attack rate was 10.6% (16/151) among exposed staff and reached 23.7% (23/97) among exposed patients in a highly vaccinated population, 16–26 weeks after vaccination (median: 25 weeks). All cases were linked and traced to one patient. Several transmissions occurred between individuals wearing face masks. Fourteen of 23 patients became severely sick or died, raising a question about possible waning immunity.

Israel was one of the first countries to achieve a high level of full vaccination with the Comirnaty (BNT162b2 mRNA, BioNTech-Pfizer, Mainz, Germany/New York, United States (US)) vaccine against severe acute respiratory syndrome coronavirus 2 (SARS-CoV-2). From May through mid-June 2021, with more than 55% of the population fully vaccinated, new cases decreased to less than two cases per million, with no social restrictions, indicative of very high vaccine effectiveness [1,2]. Since mid-June, a sharp increase in cases has been observed, attributed to the SARS-CoV-2 Delta variant (Phylogenetic Assignment of Named Global Outbreak (Pango) lineage designation B.1.617.2 and AY.* sublineages), which by mid-July constituted more than 95% of sequenced virus isolates in Israel [3]. This variant was assessed to have higher transmissibility than the Alpha variant (B.1.1.7 and Q.* sublineages) [4].

We present an investigation of a coronavirus disease (COVID-19) outbreak that started from one unidentified COVID-19 patient, with extensive, rapid nosocomial spread among vaccinated, including individuals wearing surgical masks.

## Setting 

Meir Medical Center has 780 beds, most rooms accommodate three to four patients, 1 m apart with separation curtain partitions between beds. Starting in March 2020, patients have been encouraged to wear surgical masks. Although use was inconsistent, it was enforced during patient–staff encounters for both sides. On the dedicated COVID-19 ward, dedicated staff members worked with full personal protective equipment (PPE): N-95 mask, face shield, gown, gloves and hair cover.

## Outbreak investigation

Contact investigations were carried out by trained infection control personnel and were initiated after suspected nosocomial acquisition or COVID-19 diagnosis of a staff member confirmed by positive PCR for SARS-CoV-2. All exposed individuals were PCR-tested for SARS-CoV-2. All those testing positive were considered as a COVID-19 case. All data were collected in real time and included all patients and personnel exposed to a case, last negative SARS-CoV-2 test, presence of symptoms, date of symptom onset, any sick family member, and vaccination status and date. All exposed individuals were PCR-tested for SARS-CoV-2. Whenever more than one patient was identified as COVID-19 case, all staff and patients on the ward were screened regardless of a known encounter with the positive case. All exposed patients found negative in the first screening, were cohorted and rescreened 7 days post exposure. All identified cases were either transferred to a dedicated COVID-19 unit or discharged as per clinical status. 

The index case was a fully vaccinated haemodialysis patient in their 70s. They were admitted to Ward A in mid-July with fever and cough and placed in a room with three other patients. On admission day, the index case was not tested for SARS-CoV-2, because their symptoms were mistaken for possible bloodstream infection exacerbating congestive heart failure. During their stay, the index case and one roommate were dialysed every other day in the dialysis unit. Four days after admission, the index case was diagnosed with COVID-19 by PCR for SARS-CoV-2 E gene with a quantitative cycle (Cq) value of 13.59; the case was therefore transferred to a COVID-19-dedicated unit of Ward B. On the same day, all three of this case's roommates on Ward A were screened for SARS-CoV-2 and tested positive and were transferred to the dedicated ward or discharged.

The contact investigation included Ward A, the dialysis unit (contacts of the index case) and Ward C following a 1-day stay of Case 1. This investigation revealed a total of 27 COVID-19 cases by SARS-CoV-2 PCR: 16 patients, including the index case, nine staff and two family members. 

The COVID-19 diagnosed cases were transferred on the day of their diagnosis to a COVID-19 unit on Ward B, which operated as a mixed ward because of the small number of COVID-19 patients in our hospital at the time. Half the ward was dedicated to COVID-19 patients, with dedicated staff in full PPE, while half remained a regular ward. The index case was treated on transfer day by a healthcare worker (HCW) who had recovered from COVID-19 a year earlier, and was vaccinated once, as per Israeli guidelines [5]. Three days after transfer day, this HCW attended a room in the regular ward with three patients of whom two developed symptoms compatible with COVID-19 2 days later and tested positive for SARS-CoV-2. Contact investigation on Ward B identified a total of 19 COVID-19 cases by SARS-CoV-2 PCR: 10 staff, including the aforementioned HCW, eight patients, including the three above, and one family member.

The calculated attack rate among all exposed patients and staff was 10.6% (16/151) for staff and 23.7% (23/97) for patients, in a population with 96.2% vaccination rate (238 vaccinated/248 exposed individuals).

## Sequencing and analysis

Sequence and patient data were obtained via the Israel National Consortium of SARS-CoV-2 sequencing. FASTQ files underwent processing, mapping to the reference genome (NC_045512.2) and construction of consensus FASTA sequences as previously described [6]. All sequence data were deposited and are available in GISAID [7]. Phylogenetic trees were constructed using NextStrain’s Augur pipeline and visualised with auspice [8].

We conducted phylogenetic analysis on the whole-genome SARS-CoV-2 sequences that were available for 12 cases in this outbreak, including staff and patients from Wards A, B and C and dialysis departments (Figure). All were infected with the Delta variant and epidemiologically and phylogenetically connected to the same outbreak except for Case 11 from Ward C. Case 11 and three staff members identified on Ward C were not considered as part of this outbreak. The three staff members from Ward C were exposed to both Case 1 and Case 11 and therefore the source of their infection could not be verified.

**Figure:**
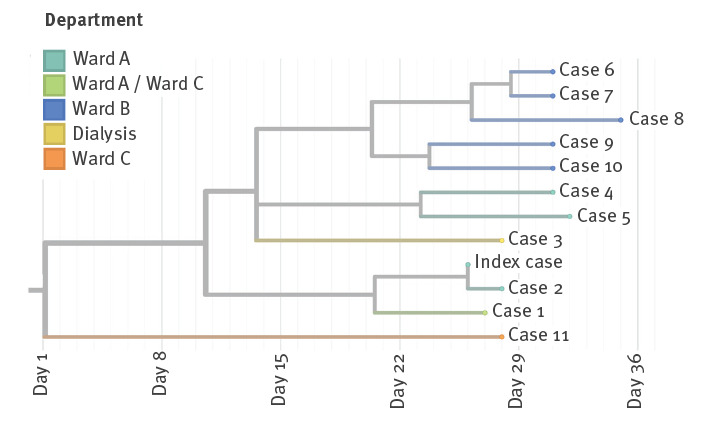
Whole genome-based phylogenetic tree of SARS-CoV-2 Delta isolates, nosocomial outbreak, Israel, July 2021 (n =12)

The tree was constructed using Nextstrain’s Augur pipeline and visualised with Auspice [8]. The numbers represent the Patient numbering used in the manuscript text and Table.

## Demographic and clinical information

Of the 42 cases diagnosed in this outbreak, 38 were fully vaccinated with two doses of the Comirnaty vaccine, one was recovered with one vaccination and three were unvaccinated. The median age was 55 years (interquartile range (IQR): 36–77.5) and 24 were female. Twenty-three were patients, 16 staff members and three family members. The median time from second vaccine dose to breakthrough infection was 177 days (range 111–194). On the day of diagnosis, only 24 individuals were symptomatic, but in the following days, 36 had become symptomatic. All staff (median age: 33 years; range: 22–48) remained asymptomatic or with mild disease. Among the patients (median age: 77 years; range: 42–93; median time from second vaccine dose to infection: 176 days; range: 143-188), eight became severely ill, six critically ill and five of the critically ill died (Table). The patient population was considerably older than staff and all patients had comorbidities: diabetes mellitus (n = 9), hypertension (n = 16), ischemic heart disease (n = 12), congestive heart failure (n = 7), dementia (n = 5), body mass index > 30 (n = 8), chronic renal failure (n = 11) of whom six were on dialysis. Eight patients were immunocompromised. 

**Table:** Case data, nosocomial COVID-19 outbreak, Israel, July 2021 (n = 23)

Case	Age group (years)	Gap (days) vaccine to diagnosis	Cq	COVID-19 maximal disease severity	Died
Index	70-79	169	13.6	Critical	Yes
1	80-89	172	15	Critical	Yes
2	50-59	175	18	Severe	No
3	60-69	176	17.6	Severe	No
4	80-89	181	20.5	Severe	No
6	40-49	143	15	Moderate	No
7	70-79	182	16	Critical	Yes
9	50-59	Not vaccinated	24	Mild	No
10	80-89	171	28	Severe	No
Na	60-69	168	18.5	Severe	No
Na	70-79	182	36	Mild	No
Na	80-89	177	31.8	Severe	No
Na	70-79	187	22	Critical	No
Na	70-79	184	14	Severe	No
Na	80-89	186	21	Asymptomatic	No
Na	90-99	173	18	Critical	Yes
Na	70-79	174	38	Severe	No
Na	70-79	176	NA	Mild	No
Na	90-99	176	NA	Critical	Yes
Na	80-89	188	NA	Mild	No
Na	60-69	183	27	Asymptomatic	No
Na	80-89	Not vaccinated	NA	Mild	No
Na	50-59	152	21.3	Asymptomatic	No

Na: not applicable; NA: not available.

The median Cq values on diagnosis days were 19.9 (IQR: 17.8–25.1) and were lower for symptomatic individuals (median: 18.2; IQR: 15.7–21.7) than for asymptomatic individuals (median: 22; IQR: 18–28), but the difference was not statistically significant.

## Ethical statement

The clinical data of this work was from an outbreak investigation; thus ethical approval was waived by the Meir Medical Center Ethical committee. The bioinformatics work was conducted according to the guidelines of the Declaration of Helsinki and approved by the Institutional Review Board of the Sheba Medical Center institutional review board (7045–20-SMC). Patient consent was waived because the study used remains of clinical samples and the analysis used anonymous clinical data.

## Discussion

We have investigated a nosocomial COVID-19 outbreak involving the SARS-CoV-2 Delta variant among a highly vaccinated population. The attack rate among exposed individuals reached 23.3% in patients and 10.3% in staff, with 96.2% vaccination rate among exposed individuals. Moreover, several transmissions probably occurred between two individuals both wearing surgical masks, and in one instance using full PPE, including N-95 mask, face shield, gown and gloves.

In a recent publication by Bernal et al., the effectiveness of full vaccination with the Comirnaty vaccine against the Delta variant was high, although lower than against the Alpha variant (88% vs 93.7%) [9]. This was not the experience in Israel, with a rapid increase in cases since June 2021 despite a high vaccination rate [1].

Although reports of breakthrough infections are increasing [10-12], this communication emphasises several points. It challenges the assumption that high universal vaccination rates will lead to herd immunity and prevent COVID-19 outbreaks. This was probably true for the wild-type SARS-CoV-2 virus, but in the outbreak described here, 96.2% of the exposed population was vaccinated. Infection advanced rapidly (many cases became symptomatic within 2 days of exposure), and viral load was high. Another accepted view is that, when facing a possible mismatch between the SARS-CoV-2 variant and vaccine or waning immunity, the combination of vaccine and face mask should provide the necessary protection. Although some transmission between staff members could have occurred without masks, all transmissions between patients and staff occurred between masked and vaccinated individuals, as experienced in an outbreak from Finland [12]. We cannot rule out that protection measures were not optimally implemented, however, transmissibility in summer 2021 differs from our experiences in the previous 18 months. Whether this can be attributed to the low Cq and high transmissibility of the Delta variant is not clear. Of note, in our cases, in particular case patients, the time from vaccination was considerable. The shortest interval was 142 days (5 months), and many of our case patients advanced to severe disease. Data from Israel imply that the main reason for the increase in COVID-19 cases in summer is indeed waning immunity, and a third vaccine dose, 5 months after the second dose will possibly result in trend reversal [13,14].

## Conclusion

This nosocomial outbreak exemplifies the high transmissibility of the SARS-CoV-2 Delta variant among twice vaccinated and masked individuals. This suggests some waning of immunity, albeit still providing protection for individuals without comorbidities. However, a third vaccine dose may be needed, particularly in individuals with risk factors for severe COVID-19. Appropriate use of masks, especially in high-risk settings is advised.
